# Auxin Extraction and Purification Based on Recombinant Aux/IAA Proteins

**DOI:** 10.1186/s12575-016-0050-1

**Published:** 2017-01-13

**Authors:** Yi Su, Weigui Luo, Xiaofei Chen, Huizhen Liu, Yueqing Hu, Wanhuang Lin, Langtao Xiao

**Affiliations:** 1 Hunan Provincial Key Laboratory of Phytohormones and Growth Development, Hunan Agricultural University, Changsha, China; 2 Hunan Co-Innovation Center for Utilization of Botanical Functional Ingredients, Changsha, China

**Keywords:** IAA, Extraction and Purification, Binding Capability, Aux/IAA, SPE

## Abstract

**Background:**

Indole-3-acetic acid (IAA) extraction and purification are of great importance in auxin research, which is a hot topic in the plant growth and development field. Solid-phase extraction (SPE) is frequently used for IAA extraction and purification. However, no IAA-specific SPE columns are commercially available at the moment. Therefore, the development of IAA-specific recognition materials and IAA extraction and purification methods will help researchers meet the need for more precise analytical methods for research on phytohormones.

**Results:**

Since the AUXIN RESISTANT/INDOLE-3-ACETIC ACID INDUCIBLE (Aux/IAA) proteins show higher specific binding capability with auxin, recombinant IAA1, IAA7 and IAA28 proteins were used as sorbents to develop an IAA extraction and purification method. A GST tag was used to solidify the recombinant protein in a column. Aux/IAA proteins solidified in a column have successfully trapped trace IAA in aqueous solutions. The IAA7 protein showed higher IAA binding capability than the other proteins tested. In addition, expression of the IAA7 protein in *Drosophila* Schneider 2 (S2) cells produced better levels of binding than IAA7 expressed in *E. coli*.

**Conclusion:**

This work validated the potential of Aux/IAA proteins to extract and purify IAA from crude plant extracts once we refined the techniques for these processes.

**Electronic supplementary material:**

The online version of this article (doi:10.1186/s12575-016-0050-1) contains supplementary material, which is available to authorized users.

## Background

As one of the most important categories of phytohormones, auxin contributes to virtually all aspects of plant growth and development [1, 2]. Thus, phytohormone quantification is very important. Currently, IAA is extracted from plant tissues and then highly purified using standardized protocols [3, 4]. Exposure to light, heat and oxygen can cause the degradation of IAA during sample preparation and purification, as IAA is not very stable in an aqueous environment. Thus, an IAA tracer labelled with radioactivity is used to estimate the amount of recovery and correct for any loss of IAA [5]. In the classical method, IAA extraction from plant tissues involves liquid nitrogen freezing/lyophilization, grinding/homogenization, treatment with organic solvents and the removal of solid impurities. IAA is a weak polar molecule. Thus, organic solvents with polarity close to IAA are the most effective for IAA extraction from plant tissues, as they result in greater efficiency and higher levels of extraction and recovery. However, a few recent studies used distilled water to extract IAA from plant samples [6]. Alternate types of extraction methods have been reported in previous studies that use various organic solvents, such as ethanol, methanol, acetonitrile, chloroform, acetone, propanol, ethyl acetate and acetic acid [7–12]. Most recently, pre-cooled 80% methanol has become a universal extraction solvent for IAA and several other phytohormones because of its high efficiency during extraction and enhanced levels of recovery [6, 13].

Purification after extraction is critical because the complex metabolites in crude plant extracts can influence the accuracy of the analysis of IAA. To overcome the interference from other compounds in the extracts, a number of methods based on different principles were applied to the extraction and purification of IAA from plant tissue. Liquid–liquid extraction (LLE) and liquid–liquid microextraction (LLME), hollow fibre-based liquid–liquid–liquid microextraction (HF–LLLME) and dispersive liquid–liquid microextraction (DLLME) have been used previously for IAA purification [14–20]. Solid-phase extraction (SPE) and antibody-based immune methods have recently become widely used to extract and purify IAA [6, 21–26]. Optimized solid-phase microextraction (SPME) and double-layered SPE (DL/SPE) resulted in a higher level of recovery and better ability to remove pigments [27, 28]. SPE and SPME cartridges filled with matrix compound (sorbent) are used to extract and purify the target molecules from mixtures in the solution, as the sorbent can selectively bind certain molecule(s) based on an array of mechanisms, including adsorption, hydrogen bonding, polar and nonpolar interactions, cation/anion exchange and size exclusion [29]. Since modern SPE/SPME techniques are usually applied online coupled with HPLC, the current emphasis involves choosing various sorbents for trapping analytes [30, 31]. Many commercial SPE/SPME columns based on different sorbents have been widely used for high-throughput phytohormone extraction and purification from crude plant extracts [22, 23, 26]. However, commercial SPE columns for multiple phytohormones, such as Sep-Pak C18, Oasis HLB, Oasis MCX and Oasis MAX, are not designed specifically for phytohormones such as IAA. Thus, they are not highly selective. Recently, molecularly imprinted polymers (MIPs) and polymer monolith microextraction (PMME) were used to improve the specific recognition capability for phytohormones [32–34]. MIPs have been widely used to detect the presence of compounds in the environment because they offer advantages such as easy, cheap and rapid preparation along with high thermal and chemical stability [35–37]. Immunoaffinity SPE sorbents, also known as immunosorbents, are based upon molecular recognition using antibodies that offer higher affinity and selectivity for the target molecule (antigen). They are suitable for extraction and purification of a single analyte from complex biological and environmental aqueous samples. Moreover, immunoaffinity chromatography (IAC) and immunoaffinity gels (IAG) have been used to purify ABA and cytokinins [38–42].

Advances in phytohormonal research require greater efficiency and increased sensitivity for the analysis of phytohormones. This should lead to advances in phytohormone extraction and purification that occur more quickly in a less complicated manner. The techniques for sample preparation are complicated, and most methods are not specially designed for IAA. In the auxin signalling pathway, auxin action is based on binding to the TRANSPORT INHIBITOR RESPONSE1/AUXIN SIGNALING F-BOX (TIR1/AFB) nuclear receptors. Auxin stabilizes the co-receptor complex composed of the TIR1/AFB and AUXIN RESISTANT/INDOLE-3-ACETIC ACID INDUCIBLE (Aux/IAA) proteins that trigger the proteasome-dependent degradation of the Aux/IAA transcriptional inhibitor to release the Auxin Response Factor (ARF) factor that induces the auxin-mediated transcriptional reprogramming [43, 44]. Interestingly, TIR1, Aux/IAA proteins and co-receptors (complex of TIR1/AFB and Aux/IAA) show high affinity towards auxin both in vivo and in vitro*,* as this hormone acts as the molecular glue that complexes the TIR1/AFB and Aux/IAA proteins [45]. Their auxin-specific binding characteristics can be utilized to improve the procedures for auxin extraction and purification. Therefore, we expressed AtIAA1, AtIAA7 and AtIAA28 in *Escherichia coli* and *Drosophila* Schneider 2 (S2) cells and developed a method for IAA extraction and purification using the recombinant proteins as the recognition molecules.

## Methods

### Chemicals and Reagents

GST Sefirose™ resin, reduced glutathione and columns were purchased from the Shanghai Sangon Biotech Company (Shanghai, China). Enzymes, relative reagents and kits in gene cloning and vector construction were purchased from TransGen Biotech (Beijing, China). Stable isotope-labelled standard [^2^H_5_] IAA was purchased from Olchemim Ltd. (Olomouc, Czech Republic). HPLC-grade acetonitrile (ACN) and methanol were obtained from the TEDIA Company Inc. (OH, USA). Other common chemicals and reagents were obtained rom the Shanghai Sangon Biotech Company (Shanghai, China). Milli-Q water was used in all experiments. The buffers used in this study are shown in Table 1.

**Table 1:** Buffers used in the study

Buffer	Components	pH value
PBS	140 mM NaCl, 2.7 mM KCl, 10 mM Na_2_HPO_4_ and 1.8 mM KH_2_PO_4_	7.4
High pH buffer	0.1 M Tris-HCl and 0.5 M NaCl	8.5
Low pH buffer	0.1 M sodium acetate, 0.5 M NaCl	4.5
Exchange buffer	50 mM Tris-HCl and 10 mM reduced glutathione	8.5

### Aux/IAAs Clone and Recombinant Vector Construction

Total RNA was extracted from 15-day-old *Arabidopsis* (*Arabidopsis thaliana*) by using Trizol reagent (Thermo Fisher Scientific Inc.) Reverse transcription was performed by using Script First-Strand cDNA Synthesis Super Mix Kit (TransGen Biotech, Beijing). Four pairs of primers were designed for recombinant vector construction based on the coding sequences of IAA1, IAA7 and IAA28 in *Arabidopsis* (Table 2). The amplification of full-length target genes was catalysed by high-fidelity DNA polymerase FastPFU (TransGen Biotech, Beijing) using cDNA as the template. The PCR products produced with the first three pairs of primers were inserted into the plasmid pGEX-KG after double digestion with *Sma*I/*Bam*HI and *Eco*RI (Thermo Fisher Scientific Inc.). *TAC* promoter drove the expression of the target genes in *E. coli*. The PCR products were inserted into pIEx-3 by using IAA7-SF and IAA7-SR after their digestion by *Sal*I and *Not*I (Thermo Fisher Scientific Inc.). IAA7 expression was driven by the *IE1* promoter with the *hr5* enhancer.

**Table 2:** Primer lists in vector constructions

Primer name	Sequences	Enzyme site
IAA1F	5′-cccgggAATGGAAGTCACCAATGGGC-3′	*Sma*I
IAA1R	5′-gaattcTCATAAGGCAGTAGGAGCTTCG-3′	*Eco*RI
IAA7-BF	5′-ggatccATGATCGGCCAACTTATGAACC-3′	*Bam*HI
IAA7-BR	5′-gaattcTCAAGATCTGTTCTTGCAGTAC-3′	*Eco*RI
IAA28F	5′-ggatccATGGAAGAAGAAAAGAGATTGG-3′	*Bam*HI
IAA28R	5′-gaattcCTATTCCTTGCCATGTTTTCTAG-3′	*Eco*RI
IAA7-SF	5′-gtcgacATGATCGGCCAACTTATGAACCTC-3′	*Sal*I
IAA7-SR	5′-gcggccgcTCAAGATCTGTTCTTGCAGTACTTC-3′	*Not*I

### Culture Conditions and Recombinant Protein Expression in *E. Coli*

Expression vectors were transformed into three *E. coli* strains, including *BL21*, *Tuner* and *Rosetta,* by electroporation. Transformants were grown at 37 °C overnight in LB medium until the OD_600_ reached 0.6. The cultures were diluted in fresh LB medium using a ratio of 1:50 and then grown in liquid culture at 37 °C on a shaker until the cells reached an OD_600_ of 0.6. Finally, the bacteria underwent another 5 h of shaking in culture to induce expression of the recombinant protein at 25 °C after adding IPTG to a concentration of 0.4 mM. The cells were collected by centrifugation and stored at −80 °C.

### Culture Conditions and Recombinant Protein Expression in S2

*Drosophila* Schneider 2 (S2) derived from a primary culture of late stage *Drosophila melanogaster* embryos was purchased from Thermo Fisher Scientific Inc. (Catalogue no. R690-07). To increase the cell yield, the S2 cells were grown in Schneider’s *Drosophila* medium (Catalogue no. 11720-034) at 28 °C without CO_2_ in suspension with spinners and shake flasks according to current protocols. Split cells at a 1:2 to 1:5 ratio were diluted into new culture every 3 to 4 days when the cells reached a density of 2 to 4 × 10^6^ cells/mL. This procedure maintained the S2 cells. The insect expression vector was stably cotransfected into S2 with pCoHygro using the calcium phosphate transfection method, and the transfectants were selected by 300 μg/mL hygromycin-B. Cell death was verified by Trypan blue staining. A 19:1 (w/w) ratio of expression vector to selection vector was used in co-transfection. Target recombinant protein was secreted into the medium and then collected by centrifugation for further purification.

### Recombinant Protein Isolation and Purification

Bacterial cells were collected and resuspended in PBS (pH 7.4). Cells were disrupted by ultrasonification. Supernatant was collected for target protein purification after centrifugation at 20,000 g for 10 min at 4 °C. For insect expression, the medium was collected from 100-mL cultures by centrifugation, since the recombinant protein was secreted into the medium. Supernatant was concentrated to less than 5 mL by centrifugation at 7500 g for 10 min in Amicon Ultra-4 Centrifugal Filter Devices with a 50 kDa nominal molecular weight limit (NMWL) from Millipore at 4 °C. Condensed culture medium was diluted 5 times in PBS to prepare it for protein purification. Protein solution was added to a column pre-filled with GST Sefirose™ resin at a speed of 1 mL/min. The purified target proteins remained trapped in the column after it was washed with 5 resin volumes of PBS.

### IAA Quantification by LC-MS/MS

Solution containing IAA was pumped into the column, and the IAA was trapped by the recombinant Aux/IAA proteins. Unbound molecules were washed off with 3 resin volumes of PBS. The outlet solution was collected after elution and freeze dried. The IAA sample was injected into LC-MS/MS (Shimadzu 8030 Plus) using a reverse C18 column to perform IAA determination after re-dissolving the compound in acetonitrile. Condition setting and programming for IAA detection were performed as described by Ma et al. [46].

## Results

### Expression of Aux/IAA Proteins in *E. Coli*

**Fig. 1:**
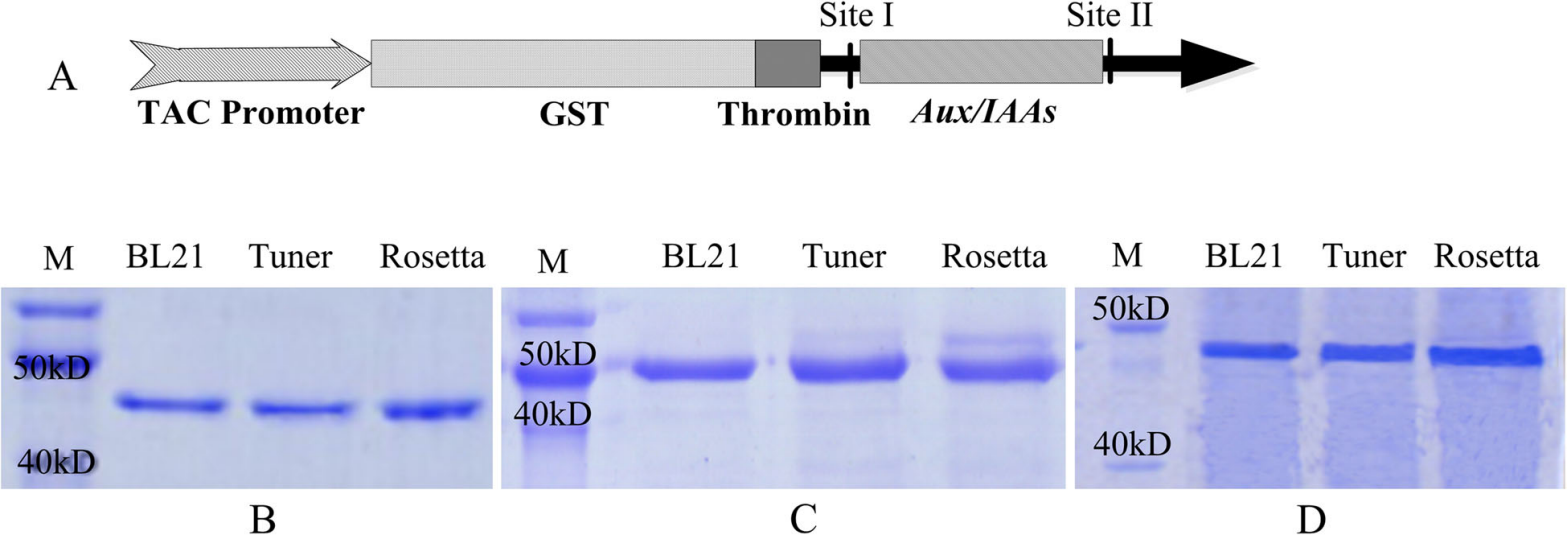
Recombinant protein expression in *E. coli*. **a** Scheme of expression vector based on pGEX-KG; **b** IAA1 expressed in strains of *BL21*, *Tuner* and *Rosetta*; **c** IAA7 expressed in strains of *BL21*, *Tuner* and *Rosetta*; **d** IAA28 expressed in strains of *BL21*, *Tuner* and *Rosetta*

In this research, the recombinant vector was constructed based on the expression plasmid pGEX-KG with a GST tag (Fig. 1a) that enabled the convenient isolation and purification of recombinant proteins using GST resin. The expression level for a particular gene in prokaryotic expression is largely dependent on the strain. To ensure that an adequate level of recombinant protein was produced, the vector containing *IAA1*, *IAA7* and *IAA28* segments was transformed into three expression strains of *E. coli,* and the expression was compared in *BL21*, *Tuner* and *Rosetta* (Fig. 1b–d). *IAA1*, *IAA7* and *IAA28* were all highly expressed in different strains when they were induced by 0.4 mM IPTG at 25 °C. The results showed that the *Rosetta* strain was more effective at expressing IAA1 and IAA28, while *Tuner* expressed the highest levels of IAA7. The cells produced 0.019 (IAA1), 0.016 (IAA7) and 0.018 (IAA28) gram of recombinant protein per gram of bacterial cells. Finally, we collected sufficient amounts of the recombinant proteins of IAA1, IAA7 and IAA28 from the cell supernatant using GST resin. No foreign bands existed after PAGE analysis (Fig. 1b–d), indicating that the highly purified protein can be used to analyse IAA binding.

### Expression of IAA7 Protein in S2 Cells

**Fig. 2:**
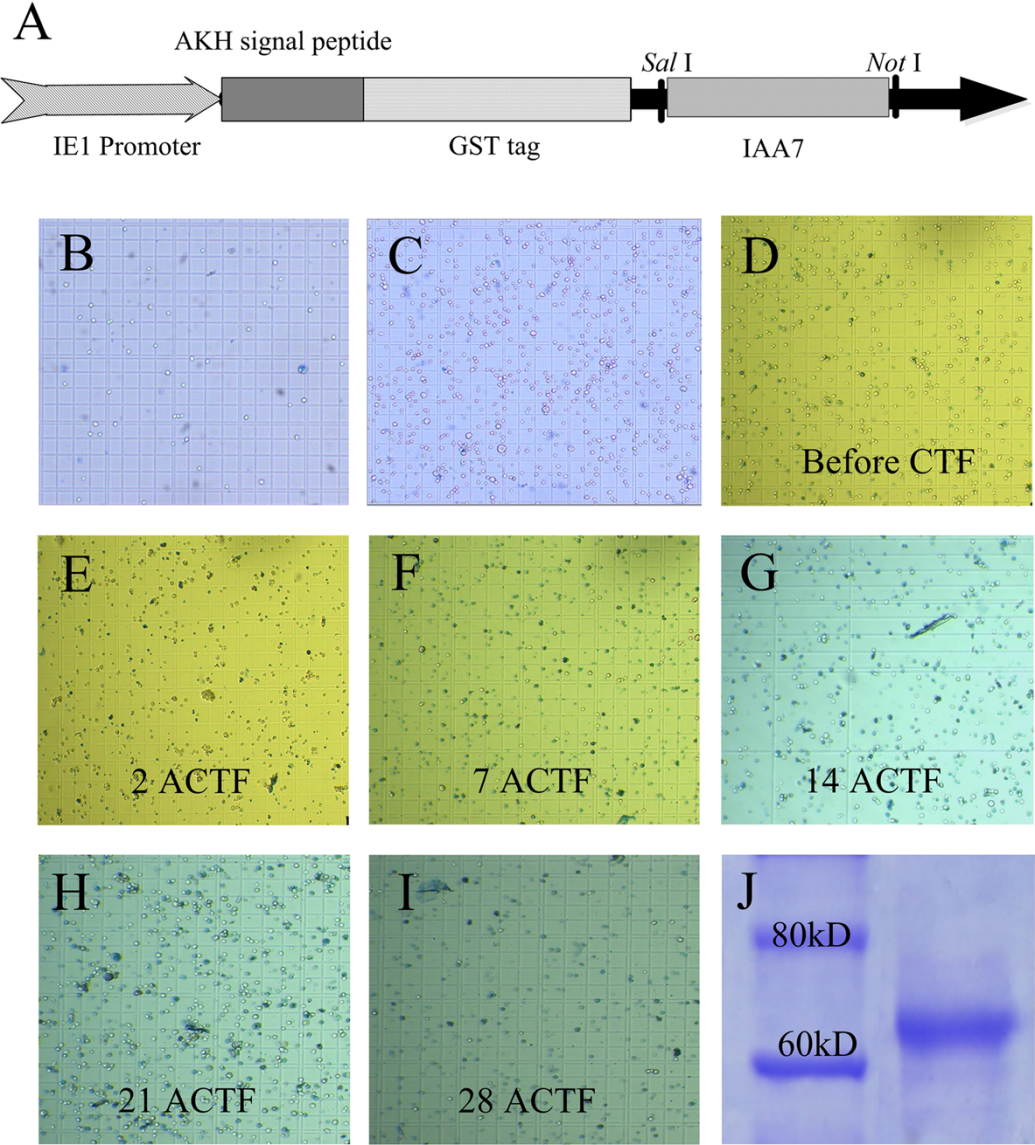
Expression of recombinant IAA7 protein in S2. **a** Scheme of expression vector based on pIEx-3; **b** 7 d adherent culture after passage; **c** 7 d suspension culture after passage; **d** S2 cells before co-transfection; **e**–**i** Stable cell line screening through homomycin after co-transfection (ACTF); **j** Gel analysis of target protein after purification from cell culture medium

In addition to prokaryotic cells, yeast, insect, mammalian and plant cells are frequently used to express recombinant proteins. Differences in protein processing, modification and folding after translation in different expression systems may influence the activity of the recombinant protein. To clarify whether the expression system affects the IAA binding characteristics of recombinant IAA7 protein, we optimized the culture conditions of S2 to be more suitable for the expression of IAA7 (Fig. 2b–j). The S2 cell line was derived from a primary culture of late-stage (20–24 h old) *Drosophila melanogaster* embryos. Many features of the S2 cell line suggest that it is derived from a macrophage-like lineage [47]. S2 cells can grow at room temperature without CO_2_ as a loose, semi-adherent monolayer in tissue culture flasks and in suspension culture with spinners and shake flasks. In our experiment, the S2 cells grew better in spinners (Fig. 2b and c). *Drosophila* Schneider 2 cells can be transfected with the recombinant expression vector alone for transient expression studies or in combination with a selection vector to generate stable cell lines. We tested the expression of IAA7 protein by transient transfection before undertaking the selection of stable cell lines. The results proved the feasibility of IAA7 expression in S2. To create stable transfectants for long-term storage, increased expression and large-scale production of the desired protein, we employed the selection vector pCoHygro to perform co-transfection with pIEx-3-IAA7 (Fig. 2a) and achieved stable cell lines by screening with hygromycin (Fig. 2d–i). For the final step, 5 mg recombinant protein was purified from 100 mL culture medium through GST resin (Fig. 2j). The recombinant protein (approximately 64 kD) was a fusion protein including N-terminal GST-Tag, His-Tag and S-Tag and adiopkinetic hormone (AKH) signal peptides that helped the recombinant protein to be secreted into the extracellular medium with high efficiency (Fig. 2a). In this study, we transfected secretion vector pIEx-3 into S2 and successfully screened a stable transfectant line. The results showed that pIEx-3 functioned effectively in *Drosophila* Schneider 2 cells, and the yield of the target recombinant protein was up to 5 mg per 100 mL culture in the stable transfectant line.

### IAA Extraction and Purification

In this study, we designed an IAA extraction and purification strategy based on the use of recombinant Aux/IAA proteins. First, we pre-filled a cartridge with GST resin to selectively bind the GST-Aux/IAA fusion proteins. This gravity column permitted isolation and purification of the target recombinant protein from the cell supernatant, as GST resin showed ideal binding activity to bioactive GST fusion proteins. Next, the aqueous sample was passed through the gravity column, and the IAA was trapped by the Aux/IAA proteins. Purified IAA solution was collected and directly used to perform IAA determination by LC-MS/MS after elution. The gravity column filled with GST resin could be reused after regeneration.

**Fig. 3:**
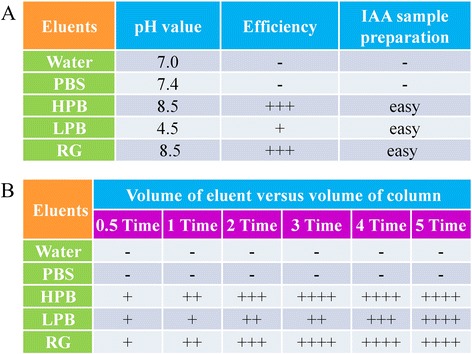
Eluting IAA through various eluents. **a** Efficiency of eluting IAA from column and difficulty level of IAA sample preparation before quantification after elution; **b** Efficiency of eluting IAA through various volumes of eluents

To obtain the greatest amount of recovery, the ideal procedure would elute all of the trapped IAA from the gravity column with a limited volume of eluant as quickly as possible. To test the efficiency of the eluants, we selected five solutions as potential eluants, including ultra-pure water, PBS, high pH buffer (HPB), low pH buffer (LPB) and GST exchange buffer (reduced glutathione, RG). Eluants of water, and particularly PBS, stabilized the protein structure and maintained bioactivity. LC-MS/MS analysis indicated that little IAA remained in solution when water and PBS (pH 7.4) were used to elute IAA from the extraction and purification column. In contrast, relatively higher alkaline or lower acid environments strongly affected the binding capability between GST and GST resin, or between Aux/IAA protein and IAA. The isoelectric points (pI) of IAA1, IAA7 and IAA28 are all greater than 7.0, thus indicating that their structures and bioactivities are more sensitive to alkaline conditions. Therefore, the results indicated that HPB (pH 8.4) could more efficiently elute the IAA from the gravity column than LPB (pH 4.5). GST exchange buffer containing 0.1 M Tris-HCl and 10 mM reduced glutathione was used to elute the expressed GST peptides from the GST resin (Fig. 3a). The eluting efficiency of the GST exchange buffer did not differ significantly from that of HPB (Fig. 3a). To simplify the process, the eluant was reduced to as small a volume as possible. In this research, IAA was almost entirely released from column after elution with 3 column volumes of HPB, LPB or RG (Fig. 3b). In this method, the outlet solution contained only a few types of molecules and ions, including IAA, recombinant protein, Tris–HCl and NaCl. Thus, the outlet solution is suitable for quantification by HPLC-MS/MS. Therefore, this method greatly facilitates the sample preparation for tandem mass spec-based IAA quantification.

**Fig. 4:**
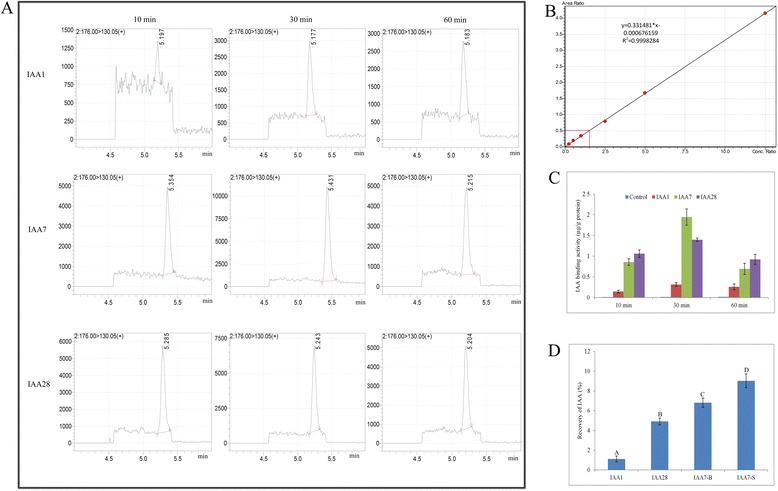
IAA extraction and purification through Aux/IAA proteins. **a** IAA determination through LC-MS/MS; **b** Standard curve of IAA; **c** IAA binding activities of Aux/IAA protein after 10 min, 30 min and 60 min incubation in column, and control was the column containing GST resin but no recombinant proteins; **d** IAA recoveries when using IAA1 (expressed in Rosetta), IAA7 (expressed in *Tuner* and S2 cells) and IAA28 (expressed in *Rosetta*) as the sorbents

The different Aux/IAA proteins showed varying affinities towards auxin. To identify the Aux/IAA protein with the highest IAA binding capability, we added 1 ng ^2^H_5_-IAA dissolved in 5 mL PBS into the gravity column containing purified GST-IAA1, GST-IAA7 or GST-IAA28 protein derived from *E. coli*. The IAA was eluted by HPB after 10 min, 30 min and 60 min incubation at 4 °C. The IAA in the outlet solution was measured using HPLC-MS/MS (Fig. 4a). The standard curve (Fig. 4b) was used to calculate the contents of IAA after different types of outlet solutions were examined (Fig. 4c). The results indicated that the incubation time was critical for IAA to bind the Aux/IAA proteins (Fig. 4c). Their binding capabilities continually decreased as the incubation time was extended. In addition, IAA7 showed higher affinity and binding efficiency for IAA when compared with IAA1 and IAA28. We also compared the difference in binding capability between IAA7 expressed in bacterial cells (IAA7-B) and that expressed in S2 (IAA7-S) (Fig. 4d). The result showed that the bioactivity of IAA7-S was significantly higher than that of IAA7-B (Fig. 4d). This result indicated that the binding capability of the recombinant protein towards IAA was higher when expressed in eukaryotes.

## Discussion

### S2-pIEx-3 System is Favourable for Expression of Higher Bioactivity Aux/IAA Proteins

The recombinant proteins derived from various expression systems displayed differing amounts of bioactivity, and the pIEx-3 was suitable for expressing highly bioactive proteins. The pIEx-3 vector was previously designed for the cloning and expression of proteins in transiently transfected *Spodoptera*-derived insect cells. Transcription is driven by the AcNPV-derived *hr5* enhancer and the immediate early promoter *IE1*. pIEx-3 contains the coding sequence for the signal peptide of adipokinetic hormone (AKH) to allow the secretion of the expressed protein [48, 49]. pIEx-3 also contains the N-terminal GST-Tag, His-Tag, and S-Tag, as well as a C-terminal HSV-Tag coding sequence for protein detection and purification (Fig. 2a). More than 40 proteins, including cytoplasmic protein kinases or regions of a receptor with kinase activity, kinase interacting proteins, phospolipases, nuclear transport proteins, phosphatases, and heat shock proteins have been expressed using this InsectDirect System, and the yields were as high as 8 mg from 100 mL culture in Sf9 and Sf21 cells [50]. In this study, the recombinant proteins displayed higher bioactivity, although lower recombinant protein yield was obtained in the stable transfectant S2 line (Fig. 4d). One potential reason for this could be that the post-translation, modification and folding in insects may differ from these processes in native plants.

### The Aux/IAA Proteins Exhibited Different IAA Affinity

Aux/IAA proteins belong to a large protein family. In *Arabidopsis*, 29 Aux/IAA proteins have been identified, and the existence of multiple Aux/IAA–ARF combinations may mediate specific responses [51–54]. The type of Aux/IAA protein may be the key factor in the auxin trapping that occurs in plant cells, since different Aux/IAA–TIR1 co-receptors varied greatly in their affinity for auxin [45]. In this study, the higher IAA affinity observed with the IAA7 protein confirms this hypothesis.

### Affinity IAA Extraction and Purification Methods Showed Great Potential

The ideal extraction and purification method for a particular phytohormone must be simple, rapid and specific to reduce its degradation and improve its recovery, as plant scientists require accurate quantification in trace plant tissues. It is critical to keep developing new extraction and purification methods for these compounds to satisfy the need for greater precision that accompanies the highly active field of investigation into the mechanisms of phytohormone actions. Currently, organic extraction and solid-phase extraction are the most frequently used methods in phytohormone extraction and purification prior to their analysis by LC-MS/MS. MIPs and some developed operational strategies, such as two-dimensional HPLC, online 2D HPLC and high-performance thin-layer chromatography (HPTLC), have been employed to purify phytohormones [23, 55, 56].

To further simplify the extraction procedure and improve specificity for a particular phytohormone, the use of bioactive protein/complex applications shows great potential in phytohormone sample preparation. For example, some functional proteins in the signalling pathway of auxin, such as auxin binding proteins, receptors and co-receptors, are able to accurately recognize and bind auxin molecules even below the fM level in plant cells. Thus, Aux/IAA proteins display a high level of affinity and selectivity towards IAA. In this study, Aux/IAA proteins were used as a selective sorbent, and an affinity IAA extraction and purification method was developed. This method eliminated the problem of interference by structurally similar compounds and simplified the procedure of IAA extraction and purification from plant crude extracts. Our application of Aux/IAA protein validated the use of phytohormonal signalling proteins in phytohormone extraction and purification.

IAA can be extracted and purified from crude sample extracts using our method, but unfortunately not all of the protein was recovered. One reason for this could be the limited bioactivity and stability of the Aux/IAA proteins. To achieve highly stable Aux/IAA proteins, other expression systems should be tested to produce recombinant proteins; other tags should be used to solidify target proteins, and other buffers should be tested for the isolation and purification of proteins and the trapping of IAA. To achieve higher binding capabilities, the IAA co-receptor, which complexes with both the TIR1 and Aux/IAA protein, should be further tested as a sorbent since this co-receptor displayed a much greater affinity for auxin [45].

## Conclusion

We expressed Aux/IAA proteins in *E. coli* and S2 cells and developed a method for IAA extraction and purification based on the recombinant proteins of Aux/IAAs. This method can be used for reference in bioactivity study and detection practice for other bioactive molecules.

## Additional file

Additional file 1: Aux/IAAs cloning and sequencing. (PDF 500 kb)

## Abbreviations

ARF: Auxin Response Factor
Aux/IAA: AUXIN RESISTANT/INDOLE-3-ACETIC ACID INDUCIBLE
CAN: Acetonitrile
DLLME: Dispersive Liquid–Liquid Microextraction
DLSPE: Double Layered SPE
HF–LLLME: Hollow Fiber-Based Liquid–Liquid–Liquid Microextraction
HPB: High pH Buffer
IAA: Indole-3-Acetic Acid
IAC: Immunoaffinity Chromatography
IAG: Immunoaffinity Gel
LLE: Liquid–Liquid Extraction
LLME: Liquid–Liquid Microextraction
LPB: Low pH Buffer
MIPs: Molecularly Imprinted Polymers
PMME: Polymer Monolith Microextraction
RG: Reduced Glutathione
SPE: Solid-Phase Extraction
SPME: Solid-Phase Microextraction
TIR1/AFB: TRANSPORT INHIBITOR RESPONSE1/AUXIN SIGNALING F-BOX
