# Supplementary material for: Auxin Extraction and Purification Based on Recombinant Aux/IAA Proteins
Source: Biol Proced Online. 2017 Jan 13;19:1. doi: 10.1186/s12575-016-0050-1 (PMC5237334; doi:10.1186/s12575-016-0050-1)
Supplement: Additional file 1: — Aux/IAAs cloning and sequencing. (PDF 500 kb) [file 12575_2016_50_MOESM1_ESM.pdf]

S1 *Aux/IAA* sequences cloned in this paper

IAA1 CDS (507 bp):
ATGGAAGTCACCAATGGGCTTAACCTTAAGGACACAGAGCTTCGTTTGGGATTACCCG
GAGCACAAGAAGAACAACAACTAGAACTTTCTTGCGTCAGAAGCAACAACAAGCGCA
AGAACAACGACTCAACAGAAGAATCTGCTCCTCCTCCTGCAAAAACACAAATCGTTG
GATGGCCTCCAGTGAGATCTAACCGTAAGAACAACAACAACAAAAACGTGAGTTATGT
GAAAGTGAGTATGGACGGAGCTCCATATCTCCGTAAGATAGATCTCAAGATGTACAAAA
ACTATCCAGAGCTTCTCAAAGCACTAGAGAACATGTTCAAGTTCACAGTAGGTGAATAT
TCCGAGAGAGAAGGCTACAAAGGATCTGGATTTGTACCTACTTATGAAGACAAAGATG
GAGATTGGATGTTGGTCGGTGATGTTCCATGGGACATGTTCTCTTCATCTTGTCAAAAA
CTCAGAATCATGAAAGGATCCGAAGCTCCTACTGCCTTATGA

IAA7 CDS (732 bp):
ATGATCGGCCAACTTATGAACCTCAAGGCCACGGAGCTCTGTCTCGGCCTCCCCGGCG
GCGCTGAAGCAGTTGAGAGTCCTGCCAAATCGGCGGTGGGAAGCAAGAGAGGCTTCT
CCGAAACCGTTGATCTCATGCTCAATCTTCAATCTAACAAAGAAGGCTCCGTTGATCTC
AAAAACGTTTCTGCTGTTCCCAAGGAGAAGACTACCCTTAAAGATCCTTCTAAGCCTC
CTGCTAAAGCACAAGTGGTGGGATGGCCACCTGTGAGGAACTACAGGAAGAACATGA
TGACTCAGCAGAAGACCAGTAGTGGTGCGGAGGAGGCCAGCAGTGAGAAGGCCGGG
AACTTTGGTGGAGGAGCAGCCGGAGCCGGCTTGGTGAAGGTCTCCATGGACGGTGCT
CCATATCTGAGGAAAGTTGACCTCAAGATGTACAAAAGCTACCAGGATCTTTCTGATGC
ATTGGCCAAAATGTTCAGCTCCTTTACTATGGGAAACTATGGAGCACAAGGAATGATAG
ATTTCATGAACGAGAGCAAGCTAATGAATCTGCTGAATAGCTCTGAGTATGTGCCAAGC
TACGAGGACAAAGATGGTGACTGGATGCTCGTTGGCGATGTCCCATGGGAAATGTTTG
TCGAGTCTTGCAAACGTTTGCGCATTATGAAGGGATCTGAAGCAGTTGGACTTGCTCC
GAGAGCAATGGAGAAGTACTGCAAGAACAGATCTTGA

IAA28 CDS (528 bp):
ATGGAAGAAGAAAAGAGATTGGAGCTAAGGCTAGCTCCTCCTTGTCACCAATTCACTT
CCAACAACAACATCAATGGATCTAAACAAAAAAGCTCGACCAAAGAAACATCATTCCT
TTCCAATAACAGGGTTGAGGTAGCTCCAGTGGTGGGATGGCCGCCGGTGAGATCATCC
CGGAGAAACCTAACGGCACAACTAAAGGAGGAGATGAAGAAGAAGGAGAGTGATGA
AGAGAAGGAATTGTACGTTAAGATCAACATGGAAGGAGTTCCAATAGGAAGAAAAGT
CAACCTTTCAGCTTATAACAACTACCAACAGCTTTCACATGCCGTTGACCAACTCTTCT
CTAAGAAAGATTCGTGGGATCTAAACAGACAATACACTTTGGTCTACGAAGACACTGA
AGGAGATAAAGTTCTGGTCGGGGATGTTCCTTGGGAGATGTTTGTATCTACTGTAAAGA
GGTTGCATGTTTTAAAGACCTCCCACGCCTTCTCACTCTCACCTAGAAAACATGGCAA
GGAATAG

S2 *IAA1* cloning and sequencing. A. PCR products of IAA1; B. Double digestion of pGEX-KG-IAA1; C. Sequencing results.

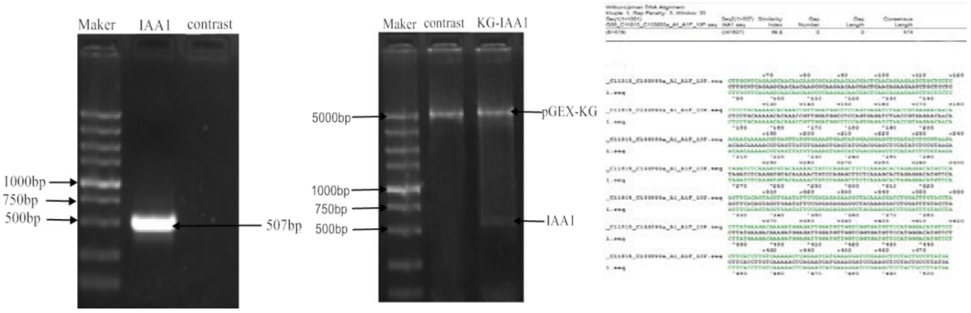

A B C

S3 *IAA7* cloning and sequencing. A. PCR products of IAA7; B. Double digestion of pGEX-KG-IAA7; C. Double digestion of pIEx-3-IAA7; D. Sequencing results.

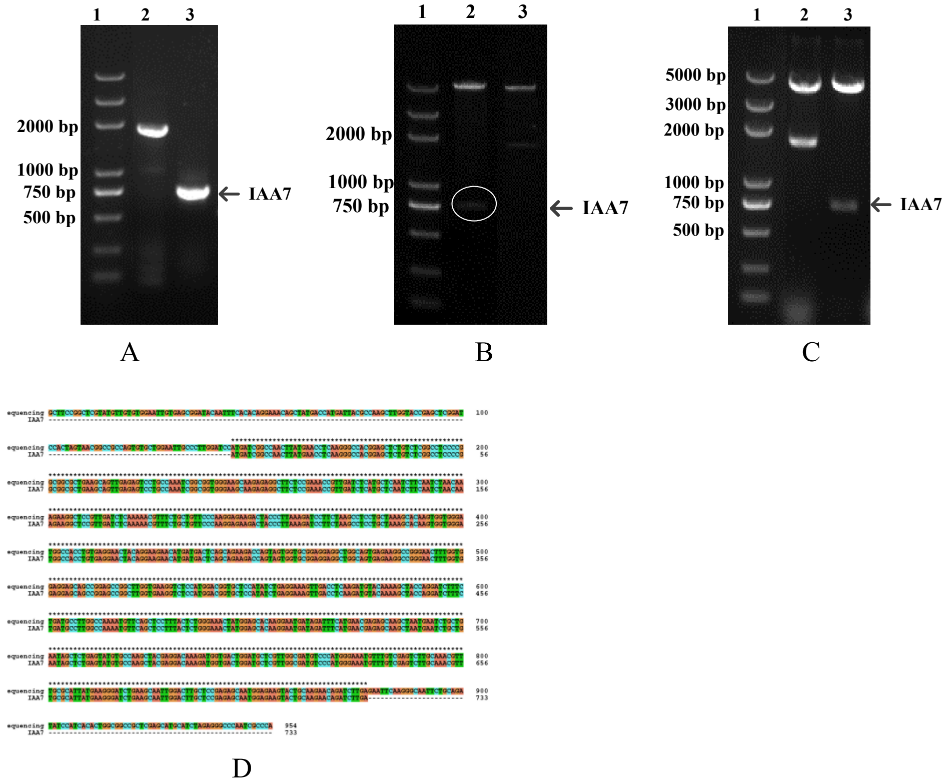

S4 *IAA28* cloning and sequencing. A. Double digestion of pGEX-KG-IAA28;B. Sequencing results.

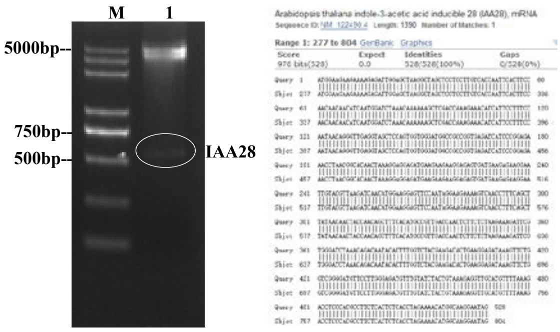

A B
